# Gross, Fine and Visual-Motor Skills in Children with Language Disorder, Speech Sound Disorder and Their Combination

**DOI:** 10.3390/brainsci13010059

**Published:** 2022-12-28

**Authors:** Cristiana Varuzza, Barbara D’Aiello, Giulia Lazzaro, Fabio Quarin, Paola De Rose, Paola Bergonzini, Deny Menghini, Andrea Marini, Stefano Vicari

**Affiliations:** 1 Child and Adolescent Neuropsychiatry Unit, Department of Neuroscience, Bambino Gesù Children’s Hospital, IRCCS, 00146 Rome, Italy; 2 Department of Human Science, LUMSA University, 00193 Rome, Italy; 3 Department of Language and Literatures, Communication, Education and Society, University of Udine, 33100 Udine, Italy; 4 Department of Life Science and Public Health, Catholic University, 00168 Rome, Italy

**Keywords:** communication disorders, linguistic assessment, preschoolers, visual-motor integration, movement assessment

## Abstract

Increasing evidence shows that children with Communication Disorders (CDs) may show gross, fine, and visual-motor difficulties compared to children with typical development. Accordingly, the present study aims to characterize gross, fine and visual-motor skills in children with CDs, distinguishing children with CDs into three subgroups, i.e., with Language Disorders (LD), Speech Sound Disorders (SSD), and LD + SSD. In Experiment 1, around 60% of children with CDs (4 to 7 years; 21 with LD, 36 with SSD, and 90 with LD + SSD) showed clinical/borderline scores in balance skills, regardless of the type of communication deficit. However, children with LD, SSD, and LD + SSD did not differ in gross and fine motor skills. In Experiment 2, a higher percentage of children with CDs (4 to 7 years; 34 with LD, 62 with SSD, 148 with LD + SSD) obtained clinical/borderline scores in Visual Perception skills. Moreover, children with LD + SSD performed significantly worsen in Visual Perception and Fine Motor Coordination skills compared to children with SSD only. Our results underlined that CDs are generally associated with gross motor difficulties and that visual-motor difficulties are related to the type of communication deficit. Paying earlier attention to the motor skills of children with CDs could help clinicians design effective interventions.

## 1. Introduction

Language development in childhood is usually associated with a variety of cognitive functions, such as working memory [1,2,3], auditory perception [4,5], and sustained attention [6,7,8]. Studies have found that motor and language development are closely related. One of the foremost pieces of evidence of the intertwined development of motor and language skills is represented by the onset of gestures during infancy [9,10]. Before mastering a language, infants usually use gestures with communicative intentionality in order to properly interact with their parents and share experiences or elicit requests. In a certain sense, motor gestures could be considered the precursors of language [9,10]. Theories of embodied cognition are in line with this theoretical background and state that the development of motor skills can influence the development of language skills, such as language comprehension [11,12].

Neuroimaging findings have also documented the intertwining of motor and language skills in the left motor and premotor areas, including the primary motor cortex [13], the ventral premotor cortex [14,15], the dorsal premotor cortex [13,14,16], the supplementary motor area, the pre-supplementary motor area [13,14], the inferior frontal cortex [17], and the cerebellum [18]. Indeed, it is well-known that language and motor skill deficits often coexist, and between 20% and 75% of children with Communication Disorders (CDs) also have a Developmental Coordination Disorder (DCD) [19,20,21].

CDs are common neurodevelopmental disorders, with an estimated prevalence of around 8% [22], characterized by delays in speech and language development in the absence of mental or physical impairment, hearing loss, emotional problems, or environmental deprivation [23]. CDs include Language Disorder (LD), which consists of reduced vocabulary and difficulty in understanding and constructing grammatical structures, and Speech Sound Disorder (SSD), which is a persistent difficulty in the production of speech sounds that interferes with speech intelligibility, as well as their combination [23].

Other CDs are the Childhood-Onset Fluency Disorder (Stuttering), the Social (Pragmatic) Communication Disorder, and the Unspecified Communication Disorder. Hereafter, we will mention CDs referring to LD, SSD, and LD + SSD only.

Epidemiological studies documented a prevalence of LD around 3.3% (aged 3 to 17 years) [24] and a prevalence of SSD between 8–9% [25]. Instead, the prevalence of the comorbid LD in patients with SSD is wide, ranging from 11 to 77%, probably due to differences in the criteria diagnosis of LD and SSD, in the instruments used during an assessment, and in the age at which children were evaluated [24]. In particular, it has been found that the comorbid LD + SSD is more common in males than in females [26], with a greater impact on individuals’ functioning in terms of learning difficulties, poorer school achievement, severe deficits in all measures of language and literacy compared to SSD only or control groups [27,28].

In children with CDs, it has been shown the presence of both fine motor deficits in activities that require muscles of the fingers, and gross motor deficits, in activities involving whole-body movement [29].

Among fine motor deficits, reduced performance was found in tasks that required planning and sequencing, such as grasping and building towers with blocks.

Specifically, a study aimed to test whether 32 children with SSD aged from 2 to 5 years had impairment in fine motor performance, assessed using the Peabody Developmental Motor Scales [30]. Findings showed that children with SSD presented difficulties in copying as well as deficits in cutting [31]. In line with these results, another study aimed to examine the comparison of gross and fine motor skills of 14 children with LD and 14 typically developing children aged from 6 to 8 years, using the Bruininks–Oseretsky motor task [32]—a task that investigates balance, bilateral coordination, upper limb coordination, visual-motor control, and upper limb speed and dexterity. Results showed that LD presented poorer performance on gross and fine motor skills than the age-matched control group [31,33].

Similarly, visual-motor skills were found to be compromised in children with LD or SSD. For example, a study found that visual-motor integration, Visual Perception, and Fine Motor Coordination skills, assessed by the Developmental Test of Visual-Motor Integration (VMI) [34], were markedly compromised in a sample of 34 children with SSD aged from 5 to 6 years compared to age-matched controls [35]. Another study investigated motor skills in 100 children and adolescents aged 4 to 14 years, of whom 56 with LD and 44 with LD + SSD. Results demonstrated poor visual-motor skills, as showed by clinical scores on the VMI, in 52% of the participants with severe LD with or without concomitant SSD [36].

Among gross motor deficits, it has been demonstrated that out of 125 children, 51% obtained borderline or clinical scores on the Movement Assessment Battery for Children [37], used to assess children’s motor skills and that children with LD performed better than children with LD + SSD and with SSD (which did not differ) [38].

Although the growing knowledge of the intertwined impairments of motor and language skills in children with CDs, a few methodological problems need to be addressed. First, the motor skills of children with CDs have so far been investigated in most studies in small groups [31,33,35], and only one study is characterized by a large sample size of around 125 children [37].

Second, in the considered studies, the relationship between language and motor aspects has been documented in groups of children with a heterogeneous and wide age range (e.g., from 24 months to 6 years), and this may have influenced previous results. In fact, age is a factor that affects the development of motor skills. Accordingly, the motor skills of a 2-year-old child are different from those expected at 6 years of age. Furthermore, it should be considered that as children grow, the demands of the environment (e.g., self-care and school work) gradually increase, and motor skills become an important means by which children interact with the external environment. Deficits in this area can, therefore, differently impact children’s functioning, including also the development of language, depending on their age [39].

Starting from these considerations, the present study aims to investigate whether children with CDs, with a narrow age range (from 4 to 7 years) and distinguished into subgroups according to the type of communication deficit, have different motor impairments. In particular, we compared subgroups of children with LD, SSD, and LD + SSD in gross, fine, and visual-motor abilities.

## 2. Materials and Methods

### 2.1. Clinical Eligibility Assessment

The cohort was retrospectively selected through a comprehensive database made of several hundred outpatients assessed at the Child and Adolescent Neuropsychiatry Unit of the Bambino Gesù Children’s Hospital (Italy).

The clinical diagnosis of CDs (LD, SSD, LD + SSD) was made according to the Diagnostic and Statistical Manual of Mental Disorders, 5th ed. (DSM-5) criteria [23] based on the developmental history, extensive clinical examination, and language assessment by expert developmental psychiatrists, neuropsychologists, and speech therapists.

To evaluate cognitive level, the brief-IQ scales from the Leiter International Performance Scale—Third Edition (Leiter-3) [40]—were administered. Language skills were assessed via the Batteria per la Valutazione del Linguaggio in bambini dai 4 ai 12 anni (BVL 4–12) [41], including lexical processing (i.e., Naming and Lexical Comprehension subtests, respectively), grammatical processing (i.e., Sentence Repetition and Grammatical Comprehension subtests), and articulation processing. In Naming subtest, children were asked to name a series of stimuli depicted in sheets. In Lexical Comprehension subtest, children were asked to point to a series of black and white target pictures after experimenter pronounced a word illustrating one of the four pictures. For each correct answer, one point was assigned. In the Sentence Repetition subtest, children were asked to listen to a sentence and then repeat the sentence they heard. In the Grammar Comprehension subtest, after hearing each stimulus sentence (e.g., “the girl pushes the boy”), children were shown a sheet with four pictures: one represented the meaning of the target sentence, whereas the remaining three pictures represented grammatical distractors (e.g., “the girl pushes the boys”, “the girls push the boy”, or “the girls push the boys”, respectively). Children were asked to point to the picture target. One point was assigned for each correct answer. In each subtest, one point was assigned for each correct answer. The total raw score was converted into standard deviation scores.

For both Experiment 1 and 2, participants’ anonymity and data confidentiality were ensured. All procedures performed in the study involving human participants were in accordance with the 1964 Declaration of Helsinki and its subsequent amendments or comparable ethical standards.

### 2.2. Experiment 1

#### 2.2.1. Participants and Procedures

The cohort was composed of 147 Italian-speaker children (Females, F/ Males, M = 30/117), including 21 with LD, 36 with SSD, and 90 with the combined disorders.

Criteria for inclusion in the study were the followings: (1) Clinical diagnosis of CDs (for details, see Table 1); (2) non-verbal intelligence quotient (nvIQ) above 85; (3) age range from 4 to 7 years included; (4) not having medical/neurological disorders (e.g., blindness, deafness or genetic syndromes); (5) not presenting comorbidities with other neurodevelopmental disorders, such as Attention Deficit Hyperactivity Disorder or Autism Spectrum Disorder, except with DCD.

**Figure 1:**
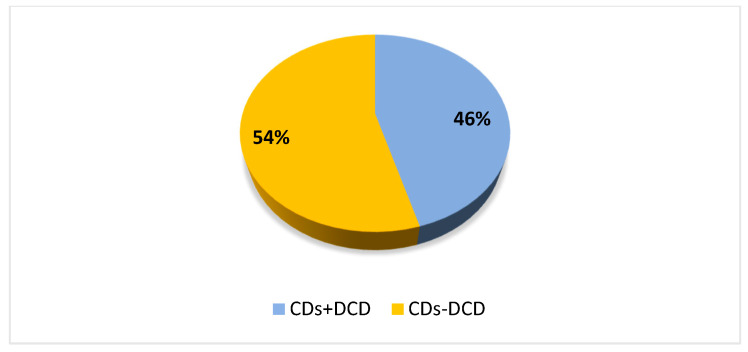
The percentage of children with CDs and a comorbid DCD regardless of subgroups. Legend: CDs + DCD = children with CDs and DCD; CDs-DCD = children with CDs without DCD.

Figure 1 presents the percentage of children with CDs and comorbid DCD regardless of subgroups. Specifically, 42.9% of children with LD also presented DCD, 38.9% of participants with SSD also had DCD as well as 48.9% of children with LD + SSD.

#### 2.2.2. Measures

##### Gross and Fine Motor Skills

The Movement Assessment Battery for Children 2 (MABC-2) was used to assess gross and fine motor skills [37]. This battery, intended for assessment of children aged 3–16 years, consists of 8 subtests that produce a Total Score and 3 component scores: Manual Dexterity, Aiming and Catching, and Balance. Manual Dexterity component evaluates fine motor skills and consists of three tasks: a one-hand posting task, a timed bimanual assembly task, and an untimed drawing task. The Aiming and Catching component aims at assessing the coordination of fine and gross motor skills and includes tasks requiring the throwing of an object to a target and the catching of an object using both hands. The Balance component assesses gross motor skills and comprises a static balance task and two dynamic balance tasks that involve sustained, controlled movement and more explosive action. For each test item, experimenters recorded designated measures (e.g., time taken to complete the task, number of successful throws/catches, and number of failures). For the Total Score and for the score of each component, the raw performance scores were then converted into standard scores (mean ± SD: 10 ± 3). The standard scores are considered problematic, i.e., clinical/borderline scores, when they are equal or below 6 (2 SDs below the mean).

### 2.3. Experiment 2

#### 2.3.1. Participants and Procedures

After clinical eligibility assessment, the selected cohort was composed of 244 Italian-speaker children (F/M = 63/181) divided into three subgroups (see Table 2): 34 with LD, 62 with SSD, and 148 with LD+. Of all children, 43 children have participated in Experiment 1.

**Figure 2:**
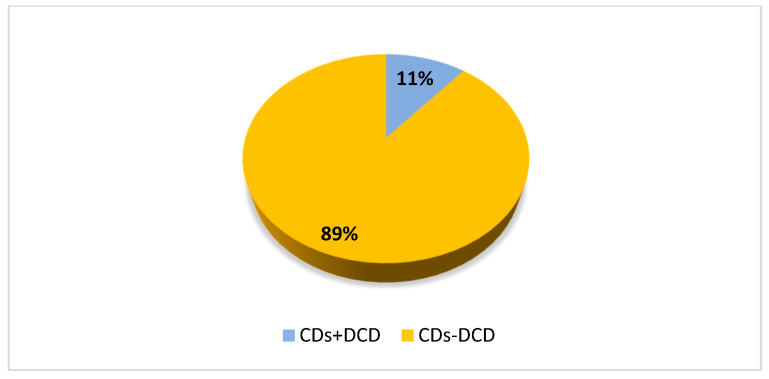
The percentage of children with CDs and a comorbid DCD regardless of subgroups. Legend: CDs + DCD = children with CDs and DCD; CDs-DCD = children with CDs without DCD.

Procedures, as well as inclusion criteria in the study, were the same as in Experiment 1. Figure 2 presents the percentage of children with CDs and comorbid DCD regardless of subgroups. Specifically, 11.8% of children with LD also presented DCD, 4.9% of participants with SSD also had DCD as well as 13.5% of children with LD + SSD.

#### 2.3.2. Measures

##### Visual-Motor Skills

The Beery–Buktenica Developmental Test of Visual Motor Integration (VMI) was used to assess visual-motor skills [42]. This test, intended for assessment of children aged from 2 years old, consists of 3 components: Visual Motor Integration, Visual Perception, and Fine Motor Coordination. The Visual Motor Integration component requires participants to imitate and copy a series of progressively more complex forms. The Visual Perception component requires children to identify matching forms when presented with similarly shaped forms, and the Fine Motor Coordination component involves children’s ability to connect dots and stay within lines of the forms. For each component, the raw performance scores were then converted into composite scores (mean ± SD: 100 ± 15) based on the normative data. The composite scores are considered problematic, i.e., clinical/borderline scores, when they are equal or below 85 (2 SDs below the mean).

### 2.4. Statistical Analysis

In Experiment 1 and Experiment 2, differences among the three subgroups (LD, SSD, LD + SSD) on chronological age and nvIQ were tested separately by two Analyses of Variance (ANOVAs), while difference in gender was verified by means of a Chi-square (χ^2^).

In Experiment 1, subgroups with CDs (LD vs SSD vs LD + SSD) were compared on the MABC-2 components (Manual Dexterity vs Aiming and Catching vs The Balance) using the Kruskal–Wallis ANOVA because the assumption of normality was not fulfilled. Multiple Comparisons *p* values (2-tailed) were applied to account for the comparisons of subgroups.

Children with LD, SSD, or LD + SSD were further classified as children with clinical/borderline scores (when standard scores on MABC-2 were equal or below 6) or children with no clinical scores (when standard scores were above 6). The percentage of children with clinical/borderline scores was calculated for each MABC-2 component (Manual Dexterity vs Aiming and Catching vs The Balance) in overall sample, regardless of the subgroups with CDs (LD, SSD, LD + SSD). Difference in the distribution of Manual Dexterity, Aiming and Catching, and The Balance within each subgroup was verified by mean of a χ^2^.

In Experiment 2, subgroups with CDs (LD vs SSD vs LD + SSD) were compared on the VMI components (Visual Motor Integration vs Visual Perception vs Fine Motor Coordination) using the Kruskal–Wallis ANOVA because the assumption of normality was not fulfilled. Multiple Comparisons *p* values (2-tailed) were applied to account for the comparisons of subgroups.

Children with LD, SSD, or LD + SSD were further classified as children with clinical/borderline scores when composite scores on VMI were equal or below 85 or children with no clinical scores when standard scores were above 85. The percentage of children with clinical/borderline scores was calculated for each VMI component (Visual Motor Integration vs Visual Perception vs Fine Motor Coordination) in overall sample, regardless of the subgroups with CDs (LD, SSD, LD + SSD). Difference in the distribution of Visual Motor Integration, Visual Perception, and Fine Motor Coordination within each subgroup was verified by means of a χ^2^.

For all analyses, a *p* value < 0.05 was considered statistically significant.

In order to evaluate a potential relation between individual factors (i.e., age, nvIQ) and motor skills, Spearman’s rank correlations (*rho*) were performed separately for LD, SSD, and LD + SSD on significant results identified by the Kruskal–Wallis ANOVA. Bonferroni’s correction was applied for multiple comparisons.

## 3. Results

### 3.1. Experiment 1

The three subgroups with CDs did not differ for chronological age (age range: 4.0–7.3 years; LD, mean ± SD: 5.28 ± 0.99 years; SSD, 5.27 ± 0.76 years; LD + SSD, 5.12 ± 0.78 years; F_2, 144_ = 0.55, *p* = 0.57), gender (LD, F/M = 5/16; SSD, F/M = 4/32; and 90 LD + SSD, F/M = 21/69; χ^2^_2_ = 2.53, *p* = 0.28) and for nvIQ (nvQI range: 81–135; LD, 102.43 ± 11.68; SSD, 102.91 ± 9.61; LD + SSD, 98.62 ± 11.17; F_2, 144_ = 2.51, *p* = 0.08).

As shown in Table 3, Kruskal–Wallis ANOVA analysis indicated that the three subgroups did not differ in each component of the MABC-2: Total (*p* = 0.14), Manual Dexterity (*p* = 0.28), Aiming and Catching (*p* = 0.43), and Balance (*p* = 0.14).

The percentage of children with clinical/borderline scores for each MABC-2 component, regardless of the subgroups with CDs (LD, SSD, LD + SSD), was 53% in Manual Dexterity, 47.6% in Aiming and Catching, and 62.6% in Balance components. No significant difference emerged across the three subgroups in the distribution of children with clinical/borderline scores (see Table 3) in Manual Dexterity (LD, 42.9.5%; SSD, 44.4%; LD + SSD, 58.9%; χ^2^_2_ = 3.18, *p* = 0.20), Aiming and Catching (LD, 47.6%; SSD, 36.1%; LD + SSD, 52.2%; χ^2^_2_ = 2.68, *p* = 0.26) and Balance (LD, 71.4%; SSD, 58.3%; LD + SSD, 62.2%; χ^2^_2_ = 0.98, *p* = 0.61).

### 3.2. Experiment 2

The three subgroups did not differ for chronological age (age range: 4.0–7.8 years; LD, 5.4 ± 0.91 years; SSD, 5.1 ± 0.78 years; LD + SSD, 5 ± 0.90 years; F_2, 241_ = 2.05, *p* = 0.13), gender (LD, F/M = 11/23; SSD, F/M = 17/45; and LD + SSD, F/M = 35/113; χ^2^_2_ = 1.20, *p* = 0.54) and for nvIQ (nvQI range: 81–137; LD, 100.05 ± 12.31; SSD, 101.01 ± 10.55; LD + SSD, 98.77 ± 11.09; F_2, 241_ = 0.93, *p* = 0.39).

As shown in Table 4, Kruskal–Wallis ANOVA analysis indicated that the three subgroups did not differ in Visual Motor Integration (*p* = 0.12). However, a significant difference emerged in the Visual Perception component among the three subgroups (*p* = 0.0015). Post hoc documented significantly worse scores in LD + SSD than in SSD (*p* = 0.004).

Moreover, a significant difference emerged in the Fine Motor Coordination component among the three groups (*p* = 0.041). Comparisons among subgroups revealed that participants with LD + SSD showed significantly worse scores in the Fine Motor Coordination component than the subgroup with only SSD (*p* = 0.039).

The percentage of children with clinical/borderline scores for each VMI component, regardless of the subgroups with CDs (LD, SSD, LD + SSD), was 16% in Visual Motor Integration, 31.6% in Visual Perception, and 28.7% in Fine Motor Coordination components. Specifically, the distribution of children with clinical/borderline scores across the three subgroups did not differ in Visual Motor Integration (LD, 17.6%; SSD, 11.3%; LD + SSD, 17.6%; χ^2^_2_ = 1.36, *p* = 0.51) as well as in Motor Coordination component (LD, 26.5%; SSD, 19.4%; LD + SSD, 33.1%; χ^2^_2_ = 4.13, *p* = 0.12). However, a significant difference in the distribution of children with clinical/borderline scores emerged across the three subgroups in the Visual Perception component (LD, 26.5%; SSD, 14.5%; LD + SSD, 39.9%; χ^2^_2_ = 13.47, *p* = 0.001).

In the LD group, significant correlations were found between nvIQ and Visual Motor Integration (*rho* = 0.39, *p* = 0.029), Visual Perception (*rho* = 0.44, *p* = 0.012), and the Motor Coordination components (*rho* = 0.45, *p* = 0.010) whereby as nvIQ increased, greater scores in VMI components were observed. No significant correlations between age and Visual Motor Integration (*rho* = 0.02, *p* = 0.87), Visual Perception (*rho* = 0.34, *p* = 0.06), and Motor Coordination components (*rho* = −0.009, *p* = 0.096) were observed.

In the SSD group, significant correlations were found between nvIQ and Visual Motor Integration (*rho* = 0.29, *p* = 0.035), whereby as nvIQ increased, greater scores in the VMI component were observed. No significant correlations between nvIQ and Visual Perception (*rho* = 0.01, *p* = 0.91) and Motor Coordination component (*rho* = 0.20, *p* = 0.12) were documented. In addition, no significant correlations between age and Visual Motor Integration (*rho* = −0.10, *p* = 0.42), Visual Perception (*rho* = 0.08, *p* = 0.61), and Motor Coordination component (*rho* = −0.008, *p* = 0.051) emerged.

In the LD + SSD group, significant correlations were found between nvIQ and Visual Motor Integration (*rho* = 0.21, *p* = 0.012), Visual Perception (*rho* = 0.29, *p* = 0.0003), whereby as nvIQ increased, greater scores in VMI components were observed. No significant correlations between nvIQ and the Motor Coordination component (*rho* = 0.05, *p* = 0.51). Moreover, a significant correlation was found between age and the Visual Perception component (*rho* = 0.18, *p* = 0.024). However, no significant correlations were found between age and Visual Motor Integration (*rho* = −0.13, *p* = 0.11) and Motor Coordination components (*rho* = 0.15, *p* = 0.07).

After Bonferroni’s correction (*p* 0.05/18 = 0.002), only the correlation between nvIQ and Visual Perception (rho = 0.29, *p* = 0.0003) survived.

## 4. Discussion

We assessed motor skills in a large group of 391 children aged 4 to 7 years according to the type of communication deficit, distinguishing between LD, SSD, and LD + SSD.

Looking at the percentages of children with clinical/borderline scores in MABC-2, around 60% obtained clinical/borderline scores in the Balance component as well as around half of the participants showed clinical/borderline in the remaining components (i.e., Manual Dexterity and Aiming and Catching). Therefore, 1:2 children with CDs—regardless of the type of deficits—may show gross and/or fine motor difficulties.

Our results are consistent with previous studies in which children with CDs tend to perform poorly on motor tasks showing difficulties in stepping, running, stair climbing, and finger gait [35,43]. Furthermore, balancing on one leg has been shown to be one of the tasks in which children with CDs have more difficulty than children with typical development [38].

Of interest, when considering gross motor skills, we observed that children with LD, SSD, and LD + SSD did not differ in MABC-2, generally obtaining clinical/borderline scores (≤6). This result further underlined that gross motor impairment is not specific to a subgroup of CDs but this aspect is compromised in children with CDs.

From a neurobiological perspective, the comorbidity between CDs and gross motor deficits could be the result of shared motor and language neuroanatomical networks [44]. Accordingly, it has been observed that cortical and subcortical regions (prefrontal cortex, basal ganglia, cerebellum) implicated in balance control are also involved in language production [45,46], and that alterations in these regions may affect motor execution and language [47].

Considering neuropsychological processes, motor and cognitive-linguistic deficits could be the result of a common procedural learning deficit [48,49,50]. The procedural memory system underlies language learning and, when impaired, gives rise to motor and language deficits that affect the acquisition of new sequential skills for word and movement production [51,52].

When considering fine and visual-motor skills, we observed that children with LD + SSD performed lower in the Visual Perception and Fine Motor Coordination components of VMI than the group with SSD alone, but not compared with the group with LD alone. Consistent with our findings, it was documented that children with comorbid LD + SSD are more impaired compared to children with SSD alone in terms of discrimination and Fine Motor Coordination (i.e., writing) [53].

Evidence demonstrated that perceptual and behavioral judgments pertaining to the auditory domain could be influenced by visual information. Specifically, auditory perceptual performance can be enhanced when the auditory information is paired with spatially and temporally coincident visual information. Conversely, pairing visual information that differs from auditory information in certain stimulus characteristics such as space, time, and semantics often results in the perception of multisensory illusions [54]. Moreover, results suggested a greater reliance on visual cues during speech perception and that visual cues can influence speech processing [55,56]. The complex interplay that occurs between the senses not only affects ongoing processing and perception but also likely plays an important role in future processes through its impact on learning and brain plasticity.

The results of the combined research suggest that there is significant facilitation of auditory comprehension and learning in the presence of salient visual cues, as well as an improvement in speech production [57,58]. The presence of clinical/borderline scores on gross motor skills and the worst performance on Visual Perception and Fine Motor Coordination in the comorbid LD and SSD compared to SSD only could mirror a greater global impairment, as supported by previous studies demonstrating a worse impact of the comorbid disorders in several areas of functioning (i.e., learning difficulties, poorer school achievement, severe deficits on all measures of language) [27,28].

However, it is worth considering that nvIQ was found to be positively correlated with performance in the LD + SSD group (higher nvIQ, better performance). Therefore, although our groups did not differ significantly in demographic variables, some aspects (for instance, nvIQ) may still have affected the results previously discussed and need to be taken into consideration for the next studies.

The co-occurrence of motor problems and language impairment may have important consequences for a child’s academic performance [27,28]. Moreover, inadequate fine and gross motor skills in children who already presented a diagnosis of CDs may further limit the child’s ability to interact socially and physically with peers, as the communication difficulties experienced by these children may negatively influence social acceptance and presumably cause them to participate less in play with peers [59,60]. Early motor skill development predicts important variability in social-communicative functioning and also in typical development [61,62].

Currently, several studies have supported the idea that rehabilitation based on audiovisual training may be the future of therapeutic interventions for individuals with CDs [54,63,64,65]. Utilizing training methods that couple meaningful, complementary auditory and visual stimuli can drastically improve performance in speech identification and learning. Indeed, speech is inherently multimodal, and redundant visual and auditory information provides salient cues about the speaker and speech content [66].

In line with the suggestions of the American Academy of Pediatrics Council on Children With Disabilities, overall results encourage screening children with CDs also for motor difficulties [67] and vice versa; children with motor impairments should be referred for a linguistic assessment. Such assessments would allow early identification of associated developmental disorders and appropriate referral to needed therapeutic services.

## 5. Conclusions

Overall, our results underlined that CDs are generally associated with gross motor difficulties and that visual-motor difficulties are related to the type of communication deficit. Thus, early identification of motor deficits and the specific characteristics of subgroups of children with CDs might help the clinicians to provide cues for effective interventions, suggesting proposing motor rehabilitation such as psychomotricity. This is a very important finding considering that many children with CDs will later develop a specific learning disorder, such as in writing.

Future research is needed to better investigate the role of motor skills in the development of CDs in order to identify the most appropriate therapy and to improve the quality of life of children and their families.

## Figures and Tables

**Table 1:** Percentages of children with LD, SSD, LD + SSD who obtained clinical scores in Naming, Comprehension, Sentence Repetition, Comprehension, and Articulation subtests (BVL 4–12).

	Lexical Processing		Grammatical Processing		Speech Processing
Groups	Naming % ^1^	Comprehension % ^1^	Sentence Repetition % ^1^	Comprehension % ^1^	Articulation % ^1^
LD	14.3	38.1	85.7	47.6	-
SSD	-	-	-	-	100
LD + SSD	40	57.8	76.7	61.1	100

^1^ The percentages refer to the number of children who showed impairment in the single subtest. Each participant may show more than one impairment in more than one subtest.

**Table 2:** Percentages of children with LD, SSD, LD + SSD who obtained clinical scores in Naming, Comprehension, Sentence Repetition, Comprehension, and Articulation subtests.

	Lexical Processing		Grammatical Processing		Speech Processing
Groups	Naming % ^1^	Comprehension % ^1^	Sentence Repetition % ^1^	Comprehension % ^1^	Articulation % ^1^
LD	29.4	44.1	64.7	38.2	-
SSD	-	-	-	-	100
LD + SSD	48	61.5	82.4	39.8	100

^1^ The percentages refer to the number of children who showed impairment in the single subtest. Each participant may show more than one impairment in more than one subtest.

**Table 3:** Mean, SD, Kruskal–Wallis H and *p*-value for Total, Manual Dexterity, Aiming and Catching, and Balance tasks by groups.

MABC-2	LD Mean (SD)	SSD Mean (SD)	LD + SSD Mean (SD)	H_(2, N = 147)_	*p*-Value
Total	5.57 ^ (2.56)	6.27 ^ (2.09)	5.34 ^ (2.49)	3.88	0.14
Manual Dexterity	6.47 ^ (2.94)	7.72 (3.93)	6.43 ^ (3.20)	2.52	0.28
Aiming and Catching	7.33 (3.91)	7.44 (2.82)	6.91 ^ (2.71)	1.67	0.43
Balance	6.04 ^ (2.33)	6.55 ^ (1.25)	5.81 ^ (2.46)	3.8	0.14

^ Clinical/borderline scores (≤−2 SDs from the mean). MABC-2 = Movement Assessment Battery for Children 2; LD = Children with Language Disorder; SSD = Children with Speech Sound Disorder; LD + SSD = Children with Learning Disorder and Speech Sound Disorder.

**Table 4:** Mean, SD, Kruskal Wallis H and *p*-value for Visual Motor Integration, Visual Perception and Fine Motor Coordination tasks by groups.

VMI	LD Mean (SD)	SSD Mean (SD)	LD + SSD Mean (SD)	H _(2, N = 237)_	*p*-Value
Visual Motor Integration	95.64 (12.31)	100.19 (14.7)	96.16 (14.10)	4.16	0.12
Visual Perception	99.22 (20.41)	100.45 (15.89)	90.79 ** (19.72)	13.03	0.0015
Fine Motor Coordination	90.71 (19.09)	94.80 (15.24)	89.63 * (14.69)	6.36	0.041

* *p* < 0.05; ** *p* < 0.01; Significant difference compared to SSD. VMI = Beery–Buktenica Developmental Test of Visual Motor Integration; LD = Children with Language Disorder; SSD= Children with Speech Sound Disorder; LD + SSD= Children with Learning Disorder and Speech Sound Disorder.

## Data Availability

The data presented in this study are available on request from the corresponding author.
